# Characterization and microRNA Expression Analysis of Serum-Derived Extracellular Vesicles in Severe Liver Injury from Chronic HBV Infection

**DOI:** 10.3390/life13020347

**Published:** 2023-01-28

**Authors:** Min Liu, Xionghao Liu, Mengmeng Pan, Yu Zhang, Xiangling Tang, Wanxi Liu, Mingri Zhao, Jing Ma, Ning Zhou, Yongfang Jiang, Wenlong Wang, Mujun Liu

**Affiliations:** 1 Center for Medical Genetics & Hunan Key Laboratory of Medical Genetics, School of Life Sciences, Central South University, Changsha 410078, China; 2 Hunan Key Laboratory of Basic and Applied Hematology, Central South University, Changsha 410078, China; 3 Hunan Key Laboratory of Animal Models for Human Diseases, Central South University, Changsha 410078, China; 4 Department of Infectious Disease, The Second Xiangya Hospital of Central South University, Changsha 410011, China; 5 Department of Cell Biology, School of Life Sciences, Central South University, Changsha 410013, China

**Keywords:** hepatitis B virus, chronic hepatitis B, cirrhosis liver decompensation, extracellular vesicles, microRNA

## Abstract

Background: Extracellular vesicle (EV) microRNAs have been documented in several studies to have significantly different expressions in hepatitis B virus (HBV)-related liver diseases, such as hepatocellular carcinoma (HCC). The current work aimed to observe the characteristics of EVs and EV miRNA expressions in patients with severe liver injury chronic hepatitis B (CHB) and patients with HBV-associated decompensated cirrhosis (DeCi). Methods: The characterization of the EVs in the serum was carried out for three different groups, namely, patients with severe liver injury-CHB, patients with DeCi, and healthy controls. EV miRNAs were analyzed using miRNA-seq and RT-qPCR arrays. Additionally, we assessed the predictive and observational values of the miRNAs with significant differential expressions in serum EVs. Results: Patients with severe liver injury-CHB had the highest EV concentrations when compared to the normal controls (NCs) and patients with DeCi (*p* < 0.001). The miRNA-seq of the NC and severe liver injury-CHB groups identified 268 differentially expressed miRNAs (|FC| > 2, *p* < 0.05). In this case, 15 miRNAs were verified using RT-qPCR, and it was found that novel-miR-172-5p and miR-1285-5p in the severe liver injury-CHB group showed marked downregulation in comparison to the NC group (*p* < 0.001). Furthermore, compared with the NC group, three EV miRNAs (novel-miR-172-5p, miR-1285-5p, and miR-335-5p) in the DeCi group showed various degrees of downregulated expression. However, when comparing the DeCi group with the severe liver injury-CHB group, only the expression of miR-335-5p in the DeCi group decreased significantly (*p* < 0.05). For the severe liver injury-CHB and DeCi groups, the addition of miR-335-5p improved the predictive accuracy of the serological levels, while miR-335-5p was significantly correlated with ALT, AST, AST/ALT, GGT, and AFP. **Conclusions:** The patients with severe liver injury-CHB had the highest number of EVs. The combination of novel-miR-172-5p and miR-1285-5p in serum EVs helped in predicting the progression of the NCs to severe liver injury-CHB, while the addition of EV miR-335-5p improved the serological accuracy of predicting the progression of severe liver injury-CHB to DeCi.

## 1. Introduction

Chronic hepatitis B (CHB) is a persistent inflammatory disease of the liver caused by hepatitis B virus (HBV) infection. Following infection with HBV, inflammation induces hepatocyte necrosis, which is a crucial pathophysiological process of disease progression. CHB will progress to severe liver damage if not managed promptly and effectively. Liver diseases constitute an enormous healthcare burden and are a growing cause of concern [1]. The World Health Organization (WHO) estimates that, in 2019, 296 million people worldwide were living with CHB. Further, hepatitis B caused approximately 820,000 deaths, mostly attributable to cirrhosis liver decompensation (DeCi) and hepatocellular carcinoma (HCC) (https://www.who.int/news-room/fact-sheets/detail/hepatitis-b, accessed on 24 June 2022). The five-year mortality rate of patients with decompensated cirrhosis without liver transplantation is up to 85% [2].

Chronic HBV infection can be classified into five phases: (I) HBeAg-positive chronic infection, (II) HBeAg-positive chronic hepatitis, (III) HBeAg-negative chronic infection, (IV) HBeAg-negative chronic hepatitis, and (V) HBsAg-negative phase [3]. HBeAg-positive chronic hepatitis B is characterized by the presence of serum HBeAg, high levels of HBV DNA, and elevated ALT. In the liver, there is moderate or severe liver necroinflammation and the accelerated progression of fibrosis [4]. Patients with compensated cirrhosis are asymptomatic or have mild clinical symptoms, with the appearance of complications with portal hypertension, ascites, hepatic encephalopathy, varicose bleeding, etc., indicating that the disease has progressed to the decompensated stage [5,6]. Serological tests, liver aspiration, and imaging can be used to help determine the course of the disease during this period. Blood tests include measurements of transaminases (ALT/AST), fibrosis-related factors, HBV DNA levels, and the presence or absence of HBV antigens [7]. They have limited effectiveness in indicating the progression of liver disease [8]. Liver biopsy is invasive and has the potential to cause serious complications, including bleeding, bile peritonitis, and pain, and it has the potential to cause diagnostic mistakes attributable to the heterogeneous distribution [9,10]. Liver stiffness with transient elastography (TE, FibroScan) and magnetic resonance elastography (MRE) are proven methods to assess liver fibrosis and cirrhosis. However, they are expensive, available only at certain limited liver health centers, and are inappropriate for patients with MR contraindications [11,12,13]. Therefore, the identification of a new non-invasive biomarker or scoring system to predict the severity of liver disease and improve current methods is urgently needed.

The presence of extracellular vesicles (EVs) with bilayer membrane structures in the circulatory system can protect target molecules and reflect the pathological or physiological conditions of the original cells or tissues [14,15,16]. EVs and their inclusions vary with physiological states and physiological conditions in the organism [17,18]. According to their biogenesis process, EVs can be further divided into exosomes, micro-vesicles, and apoptotic bodies [19]. Exosomes and the nucleic acids and proteins that they contain are regarded as types of liquid biopsies, which boast the advantages of non-invasiveness and dynamic monitoring, and they are able to overcome the limitations of tumor spatiotemporal heterogeneity [15,16]. EVs are attractive biomarkers for estimating the severity and prognosis of liver diseases, including chronic viral hepatitis B infections, cirrhosis, primary liver cancers, non-alcoholic and alcoholic steatohepatitis, and acute liver failure [20]. A study examining the potential diagnostic utility of EVs in NAFLD reported that 65 patients with NAFLD had higher concentrations of T-cell and monocyte-derived EVs than healthy controls [21]. In various studies, it has been found that several subpopulations of leukocyte-derived EVs (CD45+, CD11a+, and CD4+) are more abundant in patients with cirrhosis than in healthy individuals [22,23,24]. This may be related to the systemic inflammation of cirrhosis rather than to the severity of cirrhosis.

In this case, miRNA is a non-coding RNA molecule with a length of approximately 22 nucleotides. It inhibits gene expression by binding to the 3′ untranslated region (UTR) of a target mRNA [25]. Extracellular miRNAs are mainly secreted by specific tissues or cells and packaged into micro-vesicles, protecting miRNAs from degradation by RNases in blood [26]. EV miRNAs can act as excellent non-invasive biomarkers to evaluate disease processes, progression, and treatment responses [27,28,29]. Several studies indicated that the expression levels of some EV miRNAs in blood are significantly altered in adults with liver diseases, such as liver injury, liver cancer, and viral hepatitis [30,31]. Two miRNAs, miRNA-122 (a major hepatic miRNA) and miRNA-192, have been found to increase with fibrosis stage in serum EVs [32,33,34]. Of note, as fewer than 10 patients were included in each study, this result requires further validation. miRNA-21 expression has been found to be higher in the circulating serum EVs of patients with HCC (*n* = 30) than in patients with CHB without cirrhosis and healthy controls (*n* = 30 in each group) [35]. The expression levels of miRNA-122 in circulating serum EVs can distinguish early-stage HCC from liver cirrhosis (LC) [36]. The search for EV miRNAs that can aid in predicting whether patients with CHB will rapidly progress to DeCi disease is essential. In this study, we collected samples from patients with severe liver injury-CHB and DeCi and performed a systematic observation and a comparative analysis of the EVs and their miRNAs for both diseases to provide ideas for subsequent research. 

## 2. Materials and Methods

### 2.1. Participants and Sample Collection

A total of 58 patients with severe liver injury-CHB (HBeAg-positive chronic hepatitis B) and 33 patients with DeCi were enrolled in the present study. All patients were hospitalized in the Infectious Diseases Department of the Second Xiangya Hospital of Central South University (Changsha, China) from April 2015 to October 2020, and they were diagnosed according to the guidelines of Hepatitis B Virus-related Cirrhosis Clinical Management [37]. We established exclusion criteria to reduce the effects of other physical or pathological factors on the production of serum EV miRNAs. The exclusion criteria were as follows: patients with alcoholic cirrhosis, cirrhosis in the compensatory phases, chronic hepatitis C or D, other chronic liver diseases (such as autoimmune hepatitis, hepatolenticular degeneration, severe organ dysfunction, and hematological diseases), autoimmune diseases, malignant tumor diseases, and mental disorders. The clinical data of the patients are summarized in Table 1. We also selected 58 normal controls (NCs) who had visited the Second Xiangya Hospital of Central South University as the parallel controls. 

Blood samples were collected from all patients and healthy controls. To remove cell debris, serum separation was accomplished via centrifugation at 300× *g* for 5 min at 4 °C within 2 h, and then the samples were stored at −80 °C until the EVs separated.

### 2.2. Serum EV Isolation

The serum from 5 mL of the blood sampled from each patient was centrifuged at 1500× *g* for 10 min to eliminate cell precipitation and debris, and then the cell-free supernatant was centrifuged at 15,000× *g* for 30 min to remove large EVs. Five pools, each from the NC group and the severe liver injury-CHB group, were used for NGS sequencing, and every pool contained a mixture of samples from 5 random individuals (400 µL/sample) of the same group. For the RT qPCR analysis, 1 mL of serum was taken from each sample for detection. For NTA, TEM, and WB analyses, 0.5 mL of serum was taken for detection. Each pool or sample was filtered through a 0.22 μm pore filter (EMD Millipore, Billerica, MA, USA) to remove any larger particles. Next, each sample or pool of samples was centrifuged in a 100 KD centrifuge concentrator tube (EMD Millipore) at 3000× *g* for 30 min at 4 °C. Eventually, the supernatant from the previous procedure was enriched with 1/4 volume of ExoQuick-5A™ (System Biosciences, Inc., Palo Alto, Santa Clara, CA, USA) according to the manufacturer’s instructions. Finally, the EVs extracted were resuspended with a specific volume of phosphate-buffered saline (PBS) for different purposes and stored at −80 °C.

### 2.3. Characterization and Quantification of EVs

A nanoparticle tracking analysis (NTA) was carried out to track EV diameter and concentration. All samples were diluted with PBS before the NTA analysis, and then 100 µL of the sample was loaded into the EV analysis chamber of the Zetaview equipment PMX 110 (Particle Metrix, Meerbusch, Germany). Microsoft Excel 2013 (Microsoft Corp., Seattle, WA, USA) was used to handle the data obtained from Zetaview 8.04.02 SP2, whose parameters were optimized appropriately during the experiment.

For the transmission electron microscopy (TEM), 20 µL of the EV suspension was dropped into a carbon-coated 200 mesh copper grid in the form of a droplet for a period of time (more than 1 min). Subsequently, 2% uranyl acetate solution was added to yield negative staining, which was fixed in 10 min. The unnecessary liquid was removed using filter paper at room temperature, and then a Tecnai biological transmission electron microscope (model: Tecnai G2 Spirit, Thermo Fisher Scientific, Waltham, MA, USA) was used to obtain micrographs at 80 KV.

Further, we extracted EV proteins using RIPA Lysis Buffer (Beyotime, Shanghai, China) and a Protease Inhibitor Cocktail (Sigma-Aldrich, Darmstadt, Germany) for the Western blotting analysis. The EV proteins were then quantified using a Pierce™ BCA Protein Assay Kit (Thermo Fisher Scientific). Next, 15 µg of the EV proteins was loaded into each well of a 10% sodium dodecyl sulphate polyacrylamide gel for electrophoresis (SDS-PAGE) (Beyotime, Shanghai, China), followed by separation under the condition of 180 V (Tannon, Shanghai, China). Next, The EV proteins were transferred to a polyvinylidene difluoride (PVDF) membrane (Merck KGaA, Darmstadt, Germany) for 1.5 h with a 252 mA current. We purchased the following anti-bodies from Santa Cruz (Santa Cruz, CA, USA): anti-CD63 (sc-5275), CD9 (sc-13118), TSG101 (sc-7964), and calnexin (sc-23954). After blocking with 5% non-fat dry milk in 1 × TBST (TBS, 0.1% Tween 20) buffer for 1 h, the membrane was incubated in a primary antibody solution dissolved with 1 × TBST containing 5% non-fat dry milk (CD63, 1:2000; CD9, 1:2000; TSG101, 1:2000; and calnexin, 1:2000) at 4 °C overnight. The membrane was washed three times with 1 × TBST for 10 min. Next, the secondary antibody at a dilution of 1:10,000 was incubated at room temperature for 1.5 h and rewashed three times. The target protein was detected with an enhanced chemiluminescence kit (Super-Signal™ West Femto Maximum Sensitivity Substrate, Thermo Fisher Scientific, MA, USA) using a Bio-Rad imaging system according to the manufacturer’s instructions.

### 2.4. RNA Extraction from Serum EVs

According to the manufacturer’s instructions, the NGS sequencing sample’s total RNA was extracted from the serum EVs using an miRNeasy Mini Kit (Qiagen, Valencia, CA, USA). As for the RT-qPCR analysis samples, cel-miR-39 (RiBoBioInc., Guangzhou, China) was used as an external reference during extraction, normalizing the process of RNA extraction and PCR. Briefly, 1000 fmol of external cel-miR-39-3p was added to the EVs and mixed well. Next, the miRNA was extracted from the serum-isolated EVs utilizing a miRNeasy Mini Kit and an RNeasy MinElute Cleanup Kit (Qiagen, Valencia, CA, USA) following the manufacturer’s instructions. All RNAs were stored at −80 °C until further analysis.

### 2.5. RNA Sequencing and Data Analysis

Equal amounts of total RNA were taken for library preparation after identification and quantification using an Agilent 2100 bioanalyzer (Thermo Fisher Scientific, MA, USA) and then purified by electrophoretic separation on a 15% PAGE gel. Small RNAs of 18–30 nt were recovered and sequentially ligated to 3’ and 5’ RNA adapters. After transcription and amplification, a fragment of 110–130 bp was selected and purified. The library was quality assessed and quantitated using the Agilent 2100 bioanalyzer and QPCR, and then it was sequenced using the BGISEQ-500 platform (BGI, Shenzhen, China).

After filtering, clean tags were mapped to the reference genome with Bowtie 2 [38]. miRDeep2 was used to predict novel miRNA by exploring the secondary structure [39], and Cmsearch was performed for Rfam mapping [40]. DE miRNAs between two groups were performed using DEGseq. Next, we plotted volcano plots of DE miRNAs and the expression heatmap of 15 miRNAs online (https://www.bioinformatics.com.cn/, accessed on 20 March 2021). 

### 2.6. RT-qPCR

Stem-loop primers and specific primers for miRNAs were designed using miRNA Design software (http://www.vazyme.com/companyfile/7/, accessed on 20 March 2021), as shown in Table S1. Reverse transcription for cDNA production was performed using a HiScript III 1st Strand cDNA Synthesis Kit (Vazyme, Nanjing, China). Next, we measured miRNA levels according to the manufacturer’s instructions with miRNA Universal SYBR qPCR Master Mix (Vazyme, Nanjing, China).

In short, using RNA as a template, reverse transcription was carried out with the HiScript III 1st Strand cDNA Synthesis Kit and miRNA-specific RT primers to obtain cDNA. The cDNA was then amplified with miRNA Universal SYBR qPCR Master Mix (Vazyme, Nanjing, China) and specific primers with the following thermocycler protocol: 95 °C for 5 min + (95 °C for 10 s; 56 °C for 30 s; 72 °C for 30 s) for 40 cycles. The qPCR was run on a Bio-Rad CFX96 touch qPCR system, and a data analysis was performed using Bio-Rad CFX Manager software.

For this study, we calculated the expression levels of EV miRNAs in the samples by using the relative quantification method. For homogenization, equal quantities of cel-miR-39-3p were added as an internal control to each sample prior to RNA extraction. ΔCt was calculated as Ct (miRNA of interest) − Ct (cel-miR-39-3p), and the relative expression values for the target microRNA was calculated as 2-∆Ct. All reactions were run in triplicate.

### 2.7. Statistical Analysis

GraphPad Prism 8.3 and SPSS 23.0 were utilized for data processing and statistical analyses. After calculating whether it was a normal distribution, the differences in clinical characteristics between the severe liver injury-CHB and DeCi groups were assessed using nonparametric tests and *t*-tests, respectively. We used mean (SE) to present miRNA expression values. The differences in expression levels between the two groups were compared using the nonparametric Mann-Whitney test. A receiver operating characteristic curve (ROC) analysis was carried out to assess the predictive value of the EV miRNAs and their different combinations. The predictive values of each combination were obtained by performing binary logistic regression before the ROC curve analysis, and area under the curve (AUC) > 0.7 and *p* < 0.05 were considered the criteria for evaluating the predictive markers. Spearman’s analysis was used to evaluate the correlation between the miRNAs and clinical practices. A two-tailed *t*-test was used for *p* values, and *p* < 0.05 was regarded as statistically significant.

After calculating whether it was a normal distribution, the differences in clinical characteristics between the severe liver injury-CHB and DeCi groups were assessed using nonparametric tests and *t*-tests, respectively.

## 3. Results

### 3.1. Study Population

In this study, 149 patients (severe liver injury-CHB = 58, DeCi = 33, and NC = 58) from the Second Xiangya Hospital were enrolled. There were 25 individuals in each group (NC and severe liver injury-CHB) for NGS, and there were 33 persons in each group for training and validation. The primary characteristics and a comparison between the patients with severe liver injury-CHB and the patients with DeCi are presented in Table 1. Even though all the patients were randomly selected, there were more male patients than female patients. The vast majority of the patients with DeCi were either on or had been on antiviral therapy prior to hospital admission. The patients from the severe liver injury-CHB, DeCi, and NC groups were aged 42 (interquartile range (IQR): 29–54), 53 (IQR: 48–61), and 37 (IQR: 30.5–49) years, respectively. As expected, the patients with severe liver injury-CHB had higher levels of ALT, AST, TBIL, DBIL, GGT, and AFP than the patients with DeCi. The serum levels of WBC, L, PLT, ALB, and CHE were lower, and INR, lgA levels were higher in patients with DeCi than in patients with severe liver injury-CHB. Additionally, there were no considerable differences between the patients with severe liver injury-CHB and the patients with DeCi in other clinical features. The patients who were enrolled were not diagnosed as having alcoholic cirrhosis, hepatitis C, hepatitis D, etc.

### 3.2. Characterization of Serum EVs

**Figure 1:**
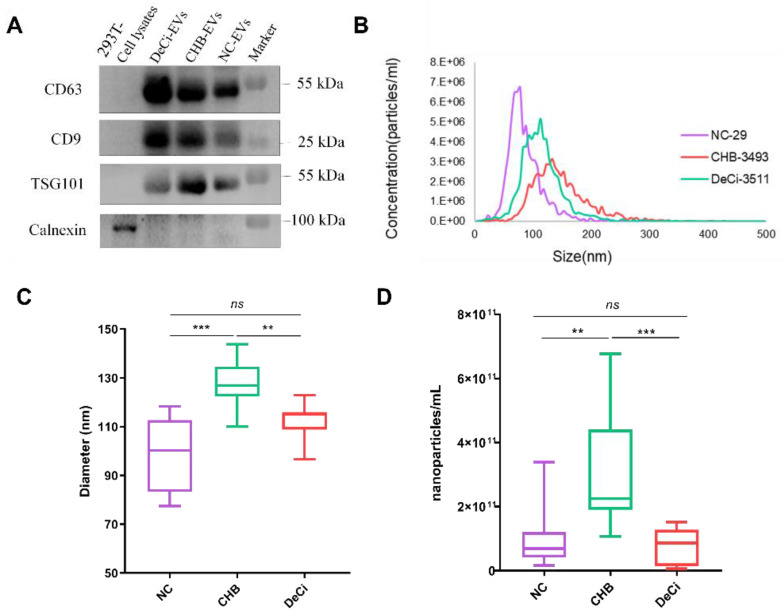
Serum-derived extracellular vesicle (EV) characterization. (**A**) Western blot analysis of common markers CD63, CD9, and TSG101 and the endoplasmic reticulum marker calnexin. Lysate of 293T cell was used as a control. (**B**) Representative size distribution of serum EVs analyzed using nanoparticle tracking analysis (NTA). (**C**) Diameter (medium) of EVs in nm determined using NTA. (**D**) Quantification of nanoparticles using NTA. In (**C**,**D**), the horizontal lines indicate the median and box, and the bars represent the 25–75th and the 5–95th percentiles, respectively. ** *p* < 0.01 and *** *p* < 0.001 (normal controls (NCs, *n* = 8), severe liver injury-chronic hepatitis B (CHB, *n* = 11), and decompensated cirrhosis (DeCi, (*n* = 8)).

We visualized the serum-derived EVs by using TEM and NTA, and then we characterized them by using a Western blot for EV markers. In the representative images, the isolated EVs with diameters of 50–150 nm and their characteristic rounded membrane-bound morphology was observed at high-magnification views (Figure S1 and Figure 1B). Next, as shown in Figure 1A, the isolated EVs displayed markedly detectable expressions of EV markers, such as CD63, CD9, and TSG101, and had no expression of the endoplasmic reticulum calnexin, which is consistent with the previously reported EV characteristics.

Additionally, in our study, the patients with severe liver injury-CHB displayed the highest EV concentrations, which were approximately 2.6-fold greater than those of the patients in the DeCi group (*p* < 0.001, Figure 1D). The EV diameter significantly differed between the NC vs. severe liver injury-CHB and severe liver injury-CHB vs. DeCi groups, with sizes of 100.23 nm [83.24–112.70], 126.77 nm [122.30–134.57], and 115.07 nm [108.78–115.95] for the NC, severe liver injury-CHB, and DeCi groups, respectively (Figure 1C). No difference in the concentrations and sizes of the EVs were found in the NTA analysis after stratification by gender (Figure S2).

### 3.3. Serum-Derived Exosome miRNA Screening

**Figure 2:**
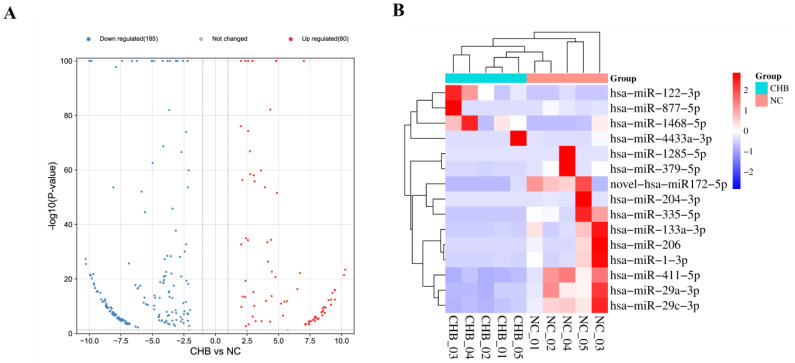
The EV miRNA expression profiles for NGS. EV miRNAs were derived from five pooled serum samples of normal controls and patients with severe liver injury-CHB. (**A**) Volcano plots of DE miRNAs (*p* < 0.05). The red dots display the upregulated expressed miRNAs in severe liver injury-CHB group relative to control group, and the blue dots display downregulated miRNAs. (**B**) Heatmap of 15 DE serum EV miRNAs (|FC| > 2, *p* < 0.0001).

We sequenced NC and severe liver injury-CHB samples in order to screen for differences in the serum EV miRNAs. First, we constructed sample pools for the different groups. For each group, we took five samples and randomly pooled them into a single pool and finally obtained five pools. The sample pools of each group were sequenced and analyzed using a high-throughput sequencing technique (NC = 25 and severe liver injury-CHB = 25). A total of 364 miRNAs were identified as differentially expressed in the sequencing, of which, 268 miRNAs were significantly up- or down-regulated (|FC| > 2, *p* < 0.05; Figure 2A). Based on sequencing results and literature, 11 miRNAs were selected for detection in our training set (|FC| > 2, *p* < 0.0001, at least ≥50 copies; Table S1). In addition, four miRNAs previously reported to be associated with disease-related molecules (hsa-miR-1285-5p, hsa-miR-204-3p, hsa-miR-335-5p, hsa-miR-877-5p) were also selected for detection (|FC| > 2, *p* < 0.0001). Heat maps of the 15 miRNAs are shown in Figure 2B. 

### 3.4. The Differences in the Expressions of miRNAs between the NC Group and the Severe Liver Injury-CHB Group

In this study, we focused on the 15 most viable candidate miRNAs for follow-up validation. Among these, miR-172-5p was a novel miRNA that was identified and sequenced (aggcuggagugcaguggcg). The primers are shown in Table S2. Ten samples each from the NC and severe liver injury-CHB groups were randomly enrolled, and serum EV RNA was extracted for an RT-qPCR assay. In all the groups screened, a total of six miRNAs were shortlisted based on valid Ct values (Ct < 35): miR-172-5p, miR-411-5p, miR-1285-5p, miR-335-5p, miR-877-5p, and miR-4433a-3p.

**Figure 3:**
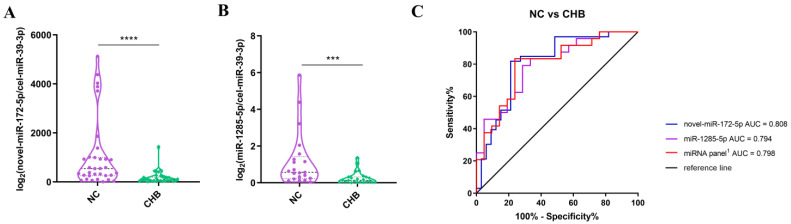
Serum EV miRNA expression levels evaluated by RT-qPCR and ROC analysis of classifiers’ efficiencies in distinguishing between NC and severe liver injury-CHB groups. (**A**,**B**) The expression profiles of serum EV novel-hsa-miR-172-5p (**A**) and hsa-miR-1285-5p (**B**) in NCs (*n* = 33) and patients with severe liver injury-CHB (*n* = 33). Each *p* value was calculated using a nonparametric Mann–Whitney test. *** *p* < 0.001; **** *p* < 0.0001; and ns, not significant. (**C**) ROC analysis of differentially expressed EV miRNAs and combinations in NCs and patients with severe liver injury-CHB. miRNA panel^1^ is the combination of miR-172-5p and miR-1285-5p.

For a further evaluation of the potential of these miRNAs, the levels of these six individual miRNAs were assessed by RT-qPCR in an independent cohort of NCs (*n* = 23) and patients with severe liver injury-CHB (*n* = 23). The miRNA levels were normalized by cel-miR-39-3p. Interestingly, only miR-172-5p and miR-1285-5p were found to be significantly differentially expressed (Figure 3A,B and Figure S3). 

In order to assess the miRNAs, we performed a receiver operator characteristic (ROC) curve analysis. Comparing the severe liver injury-CHB group with the NC group, the areas under the curve (AUCs) of miR-172-5p and miR-1285-5p were 0.808 (95% confidence interval (CI): 0.7003–0.9159, *p* < 0.001) and 0.794 (95% CI: 0.664–0.924, *p* < 0.001), respectively (Figure 3C). MiR-172-5p showed the highest sensitivity and specificity, with percentages of 84.9 and 72.7% (Table S3), respectively. Next, we evaluated the predictive values of various combinations of these miRNAs [24]. The combination of two miRNAs (miR-172-5p and miR-1285-5p) showed an AUC area of 0.798 (95% CI: 0.6661–0.9291, *p* < 0.001) (Figure 3C), and its sensitivity and specificity were 68.2% and 90.5%, respectively (Table S3). These data suggest that low expressions of miR-172-5p and miR-1285-5p in serum EVs predict a higher risk of chronic mild CHB developing into severe liver injury.

### 3.5. The Difference in the Expressions of miRNAs between the NC Group and the DeCi Group

We simultaneously examined the expressions of the six shortlisted miRNAs in the patients with DeCi (*n* = 33) and obtained results similar to those of patients with severe liver injury-CHB. Compared to the NC group, miR-172-5p and miR-1285-5p showed significantly decreased expression levels (based on the RT-qPCR results) in the DeCi group. Additionally, miR-335-5p was significantly downregulated in the DeCi group compared to that in the NC group.

**Figure 4:**
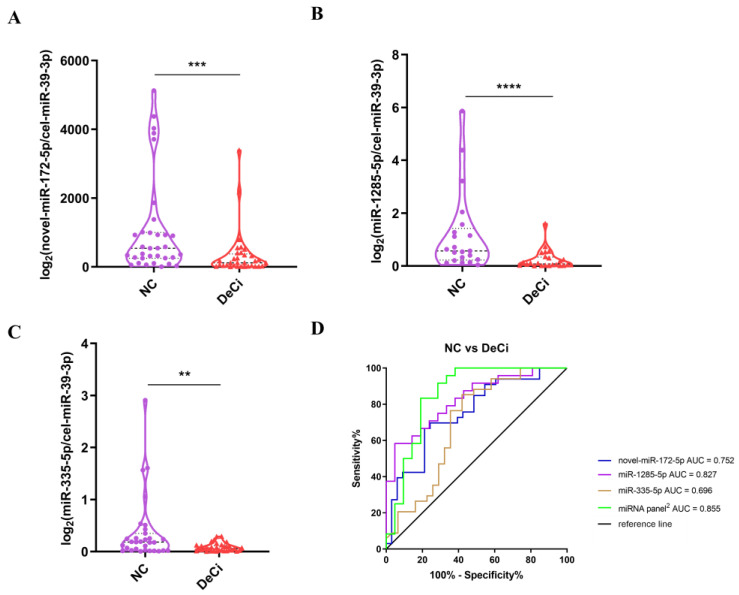
Verification of selected miRNAs in serum EVs by qRT-PCR and assessing the predictive ability of EV miRNAs to indicate the risk of severe liver injury-CHB progressing to DeCi by ROC curve. (**A**–**C**) The expression profiles of serum EV novel-miR-172-5p (**A**), miR-1285-5p (**B**), and miR-335-5p (**C**) in NCs (*n* = 33) and patients with DeCi (*n* = 33). Each *p* value was calculated with a nonparametric Mann–Whitney test. ** *p* < 0.01; *** *p* < 0.001; **** *p* < 0.0001. (**D**) ROC analysis of differentially expressed EV miRNAs and combinations in NCs and DeCi. miRNA panel^2^ is the combination of miR-172-5p, miR-1285-5p, and miR-335-5p.

The ROC curve analysis showed that the AUCs of miR-172-5p, miR-1285-5p, and miR-335-5p were 0.752 (95% CI: 0.6345–0.8697, *p* < 0.001), 0.827 (95% CI: 0.7094–0.9453, *p* < 0.001), and 0.696 (95% CI: 0.5627–0.8292, *p* < 0.01), respectively (Figure 4). The largest AUC was 0.855 (95% CI: 0.7345–0.9758, *p* < 0.0001) for a combination of three miRNAs (miR-172-5p, miR-1285-5p, and miR-335-5p) compared to those of single miRNAs or other miRNA panels. The sensitivity and specificity of this combination were 83.3% and 81.0%, respectively (Figure 4 and Table S3). This combination in serum EVs displayed a high predictive accuracy for DeCi vs. NCs.

### 3.6. The Differences in the Expressions of miRNAs between the Severe Liver Injury-CHB Group and the DeCi Group

**Figure 5:**
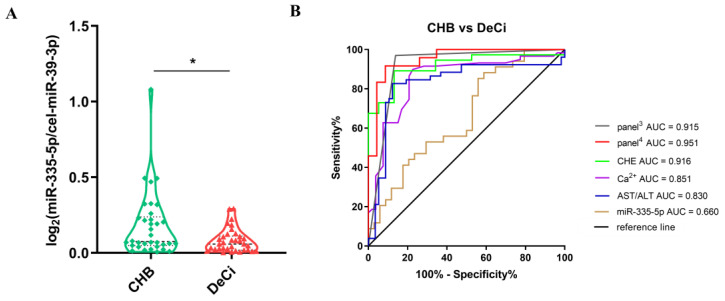
Serological expressions of EV miRNAs and ROC analysis for severe liver injury-CHB vs. DeCi groups. (**A**) The expression profiles of serum EV miR-335-5p in patients with severe liver injury-CHB (*n* = 33) and patients with DeCi (*n* = 33). *p* value was calculated by nonparametric Mann–Whitney test. * *p* < 0.05. (**B**) ROC analysis of miR-335-5p and significantly different expressed clinical indices and their combinations in CHB and DeCi. panel^4^ is the combination of AST/ALT, Ca^2+^, CHE; panel^3^ is the combination of AST/ALT, Ca^2+^, CHE and miR-335-5p.

Furthermore, we performed a statistical analysis of the data from the severe liver injury-CHB and DeCi groups, and we found that miR-335-5p was further reduced in the DeCi group compared to that in the severe liver injury-CHB group (*p* < 0.05) (Figure 5).

The ROC curve analysis of miR-335-5p showed an AUC area of 0.660 (95% CI: 0.5307–0.7893, *p* = 0.023). The low expression of miR-335-5p in serum EVs predicts a greater risk of further development of DeCi in patients with CHB and severe liver injury.

Additionally, we attempted to perform a joint analysis based on the available clinical indicators. We systematically analyzed the differences in the serological levels between the severe liver injury-CHB and DeCi groups and calculated their predictive sensitivity and specificity. The sensitivity and specificity of CHE, Ca^2+^, and AST/ALT exceeded expectations and showed better predictive properties (Table S3). The highest sensitivity and specificity (91.7% and 91.3%, respectively) were achieved after combining miR-335-5p with the aforementioned clinical indicators.

Additionally, to detect the correlation between miR-335-5p and disease, we calculated the correlation between miRNA concentrations and serological detection levels using Spearman’s correlation analysis. The levels of miR-335-5p were remarkably related to the degree of inflammation (ALT and AST), GGT, and the index of hepatocyte regeneration (AFP) in a wide range of serological parameters (Table 2). However, miR-172-5p and miR-1285-5p displayed no statistical relevance to serological levels.

## 4. Discussion

HBV infection is the primary cause of progressive liver disease. Liver biopsy has the potential to cause diagnostic mistakes attributable to heterogeneous distribution [9]. Blood tests include measurements of transaminases (ALT/AST), fibrosis-related factors, HBV DNA levels, and the presence or absence of HBV antigens. They have limited effectiveness in indicating the progression of liver disease [7]. In the past few decades, identifying biological phenotypes at the histological level has shown great potential for disease diagnosis. For example, clinical glycomic techniques used to distinguish patients with compensated cirrhosis from those with non-cirrhotic chronic liver disease have shown a sensitivity of 79% and a specificity of 86% [41]. Using genome-wide miRNA microarrays, miR-106b and miR-181b in serum were identified as specific biomarkers with an AUC of 0.774 for chronic HBV-associated liver cirrhosis (HBV-LC) and 0.915 for non-chronic HBV-LC [42]. Additionally, miR-101 in serum was reported to discriminate HBV-HCC from HBV-LC with a sensitivity of 95.5% and a specificity of 90.2% [43]. Serum microfibrillar-associated protein 4 (MFAP-4) can help distinguish patients with LC from individuals without liver disease (AUC = 0.97) using a proteomic approach [44].

EVs, as well as EV RNAs and EV proteins, are regarded as types of liquid biopsy, which boasts the advantages of non-invasiveness and dynamic monitoring, and they are able to overcome the limitations of tumor spatiotemporal heterogeneity. miRNAs are short non-coding RNA oligonucleotides that can increase mRNA degradation or decrease their translation by targeting specific mRNAs [45]. EVs with bilayer membranes in the circulatory system can protect their miRNA cargos and influence the target cells’ behavior, making EVs a popular candidate for studying the mechanisms of diseases and intercellular communication [15,16]. The role of EV miRNAs in HCC has been studied in several articles. For instance, serum exosome miR-122 levels are lower in patients with HCC than in patients with CHB or DeCi [46,47]. In contrast to normal hepatocyte-derived exosomes, 49 significantly over-expressed miRNAs have been observed in HCC [48].

In this study, EVs were isolated from serum based on their physical size and the principle that hydrophobic protein and lipid molecules can bind to PEGs. EVs were identified based on their physical shape, diameter, and protein markers, such as CD63 and CD9 [16]. In the Western blot results, we observed a gradual increase in the expressions of CD63 and CD9 with disease progression. ScRNA-seq demonstrated the expansion of macrophages in a mouse model of NASH with a pro-inflammatory phenotype, and CD9 is overexpressed in macrophages [49]. Furthermore, Sabine et al. confirmed that macrophage-mediated inflammation is critical in the pathogenesis of non-alcoholic steatohepatitis (NASH), and a higher level of CD63 and CD9 expression was detected in monocyte-infiltrating macrophages entering the liver [50]. The high expression of CD63 and CD9 found in the current research may be associated with macrophages, and more profound validation studies will be necessary shortly.

We observed that patients with severe liver injury-CHB had the largest EVs size, which became smaller when progressing to DeCi. It has been reported that the expression level of TSG101 can affect the quantity and size of EVs [51]. In the present study, we also detected higher expression levels of TSG101 in patients with severe liver injury-CHB than in patients with DeCi, which may contribute to the change in EV size. In addition, changes in the physiological environment may also affect the size of EVs. For example, increased micro-environment acidity led to significantly smaller EV sizes in patients with prostate cancer (PCA) compared to individuals with no signs of urological disease [52].

Many studies have demonstrated that higher EV concentrations are detected in patients with diseases when comparing multiple diseases with normal groups [53,54,55,56,57]. In the current study, we also confirmed that patients with severe liver injury-CHB had the most numerous EVs. Interestingly, in our study, it was also found that the Ca^2+^ levels in peripheral blood were significantly higher in patients with severe liver injury-CHB than in patients with DeCi (*p* < 0.001), with a similar trend to the concentration of EVs. It has been demonstrated that exosomes are released in a Ca^2+^-dependent manner [58,59]. The production and shedding of red blood cell (RBC)-derived MV are associated with calcium and calcium carriers [60,61]. Increased intracellular Ca^2+^ concentrations in erythrocytes and platelets promote micro-vesicular release [62,63]. In addition, Ca^2+^ influx promotes cellular repair when a certain level of cellular damage has occurred, and the size or shape of the specific membrane damage prompts its removal via lysosomal cytosolic spitting or micro-vesicular shedding [64]. We speculate that the distribution of the number of EVs in the patients in this study may have some correlation with Ca^2+^ levels.

The ROC analysis of the NC group compared with both the severe liver injury-CHB and DeCi groups indicated that the novel microRNA miR-172-5p was the best predictor, followed by miR-1285-5p. miR-335-5p might predict the risk of the further development of DeCi in patients with CHB and severe liver injury.

The novel-miR-172-5p was a newly identified miRNA in this study. miR-172-5p was predicted to target multiple disease-related genes using bioinformatics software. CAMP response element-binding protein 1 (CREB1) is a core transcription factor, and it may be a promising therapeutic target for liver diseases [65]. HBV DNA polymerase attenuates HBV replication by activating the CREB1-HOTTIP-HOXA13 axis [66]. However, in this study, Spearman’s analysis did not find it to be associated with any clinical features (such as HBV DNA). Insulin-like growth factor binding protein 5 (IGFBP5) is also noteworthy, as it induced effects similar to those induced by TGFB1 [67]. IGFBP5 is a secretory protein associated with cell proliferation, adhesion, migration, systemic inflammatory response, and fibrosis. Furthermore, IGFBP is involved in the evolution of liver fibrosis, where an increase in IGFBP5 likely leads to a higher production of collagen type I in fibroblasts and enhanced tissue fibrosis [68]. novel-miR-172-5p is likely to be a helpful marker for liver diseases. Decreased expression levels of novel-miR-172-5p may promote the liver fibrosis process by affecting its target gene IGFBP, for which a more systematic study of this view is essential.

There is no clear evidence of a correlation between miR-1285 expression levels and chronic liver diseases, such as HCV infection, non-alcoholic steatohepatitis, and autoimmune liver disease. Several papers have reported their association with tumor processes. As a regulatory miRNA of p53, miR-1285-5p is a tumor suppressor that inhibits cell proliferation and migration. One study showed that miR-1285-5p expression was lower in tumor tissues than in normal tissues [69,70]. DAPK2, a known tumor-suppressor, mediates both the anti-proliferative and the pro-apoptotic effects of miR-1285 depletion [71]. Moreover, the over-expression of miR-1285 weakens the TGF-β2-induced EMT process [72]. Considering these reports, we speculate that miR-1285-5p may be essential in regulating the development of liver parenchymal cells into fibrotic cells. More functional studies on miR-1285-5p and its targets are expected.

miR-335 regulates numerous genes and plays multifunctional roles. Through exosomes, miR-335-5p completes the transfer from donor to recipient cells and promotes the migration, invasion, and EMT of cancer cells [73]. Transcription Factor EB (TFEB), a major regulator of lysosomal biogenesis and autophagy, increases insulin receptor substrate 1 (IRS1) protein and modulates glucose tolerance by decreasing miR-335 levels [74]. Additionally, in our research, the expression of miR-335-5p was strongly correlated with the degree of inflammation (ALT, AST), GGT, and the index of hepatocyte regeneration (AFP) levels, which may facilitate an intensive study of the mechanisms of disease progression.

The diagnosis of HBV-related cirrhosis, which is the result of the development of CHB, involves the diagnosis of etiology, the assessment of compensated or decompensated status, and the evaluation of complication profiles. We observed the characteristics of the EVs in the disease phenotypes in the severe liver injury-CHB and DeCi samples in this study. The patients with severe liver injury-CHB had the highest number of EVs, which might be closely associated with Ca^2+^ concentrations. The expression level of novel-miR-172-5p in serum EVs differed significantly between the healthy individuals and the patients with severe liver injury-CHB or DeCi. The expression level of miR-335-5p decreased gradually with the progression of HBV-associated liver disease. Although the difference in miR-335-5p used in this study could not satisfactorily distinguish between the severe liver injury-CHB and DeCi groups, the inclusion of EV miRNA improved its predictive accuracy. A more comprehensive serological analysis of the different phases of HBV disease progression holds promise for predicting disease progression and for diagnosing compensated cirrhosis. Furthermore, studies on the correlation of miRNAs with liver fibrosis progression, inflammation, and hepatocyte regeneration would be greatly appreciated.

## 5. Conclusions

We successfully isolated EVs and analyzed their concentrations and miRNA components. Patients with severe liver injury-CHB had the highest number of EVs, which might be closely related to Ca2+ concentrations. Furthermore, this study revealed the differential expression profiles of serum EV miRNAs in NC, severe liver injury-CHB, and DeCi groups, and it verified the candidate miRNA expression differences in the different groups using RT-qPCR. novel-miR-172-5p and miR-1285-5p in serum EVs provided a positive predictive accuracy for severe liver injury-CHB vs. NC. A combination of three miRNAs (novel-miR-172-5p, miR-1285-5p, and miR-335-5p) on a panel had the highest detection accuracy when differentiating the DeCi group from the normal group. Additionally, miR-335-5p was statistically different compared to the DeCi and severe liver injury-CHB groups, which may indicate a positive correlation with the level of inflammation. The addition of EV miR-335-5p improved the serological level of accuracy in predicting severe liver injury-CHB progression to DeCi. In summary, our research demonstrated the differences in EV characteristics and EV miRNA expressions between NC, severe liver injury-CHB, and DeCi groups, providing ideas for subsequent studies of predictive markers and studies of disease progression mechanisms.

## Figures and Tables

**Table 1:** The characteristics of participating patients and healthy controls.

Variable	Severe Liver Injury-CHB (*n* = 58)	DeCi (*n* = 33)	*p* Value	NC (*n* = 58)
Gender (male/female)	50/8	26/7	0.23	23/35
Age (years)	42 (29, 54)	53 (48, 61)	<0.001	37 (30.5, 49)
WBC (10^9^/L)	5.37 (4.23, 6.92)	3.50 (2.42, 5.57)	<0.001	/
N (10^9^/L)	3.60 (2.66, 4.69)	2.16 (1.39, 4.30)	<0.001	/
L (10^9^/L)	1.22 (0.92, 1.58)	0.63 (0.46, 1.06)	<0.001	/
PLT (10^9^/L)	137 (113, 181)	73.00 (39.00, 109.75)	<0.001	/
ALT (U/L)	207.80 (84.05, 454.55)	28.10 (14.90, 45.75)	<0.001	/
AST (U/L)	110.00 (69.15, 171.50)	39.30 (26.93, 68.05)	<0.001	/
AST/ALT	0.57 (0.35, 1.00)	1.60 (1.29, 2.14)	<0.001	/
ALB (g/L)	34.03 ± 5.13	29.05 ± 5.12	<0.001	/
GLO (g/L)	29.80 (26.95, 34.00)	30.80 (26.70, 33.40)	0.56	/
TBIL (µmol/L)	144.50 (20.40, 201.95)	38.60 (19.80, 89.50)	0.01	/
DBIL (µmol/L)	101.50 (11.45, 163.10)	16.95 (7.90, 60.58)	0.005	/
Cr (µmol/L)	68.20 (56.45, 80.10)	71.10 (61.98, 110.53)	0.13	/
INR	1.18 (1.04, 1.43)	1.42 (1.33, 1.56)	<0.001	/
Serum calcium (mmol/L)	2.12 ± 0.09	1.98 ± 0.12	<0.001	/
CHE (IU/mL)	4524.30 (3674.00, 5855.88)	2382.30 (1336.25, 3098.20)	<0.001	/
AKP (U/L)	114.90 (75.80, 132.40)	85.90 (61.10, 131.68)	0.08	/
GGT (U/L)	114.10 (77.88, 186.53)	48.15 (21.00, 63.10)	<0.001	/
IgA (g/L)	2.77 (2.62, 4.17)	4.41 (3.29, 6.76)	0.004	/
AFP (ng/mL)	207.00 (7.54, 294.00)	2.35 (1.34, 6.92)	<0.001	/
HBeAg (±)	25/25	18/33	0.01	/
HBeAb (±)	28/23	11/39	0.43	/
HBV DNA (copies/mL)	513,000 (21,300, 11,150,000)	0.07 (0, 904)	<0.001	/

Data are shown as median and range (the smallest and largest values are in quotes) or means ± SD. WBC: white blood cell count; N: neutrophil; L: lymphocyte; PLT: platelet count; ALT: alanine aminotransferase; AST: aspartate aminotransferase; ALB: albumin; GLO: globulin; TBIL: total bilirubin; DBIL: direct bilirubin; Cr: creatinine; INR: international normalized ratio; CHE: cholinesterase; AKP: alkaline phosphatase; GGT: gamma-glutamyl transpeptidase; lgA: immunoglobulin A; AFP: alpha-fetoprotein; HBeAg: hepatitis B e antigen; HBeAb: hepatitis B e antibody; HBV DNA: hepatitis B virus DNA. Features that were not collected from NC are indicated by “/”; *p* value of <0.05 was considered significant.

**Table 2:** The results of Spearman’s rank correlations between miR-335-5p and other variables in patients with liver disease.

Variable	rs	*p*
ALT	0.449 **	<0.001
AST	0.412 **	0.001
AST/ALT	−0.296 *	0.016
GGT	0.287 *	0.04
AFP	0.397 **	0.003

‘rs’ is the correlation coefficient between serum EV miR-335-5p and other variables. A *p*-value < 0.05 was considered statistically significant; * *p* < 0.05; ** *p* < 0.01.

## Data Availability

The Gene Expression Omnibus (GEO) database accession number for the miRNA profile raw data in this study is GSE188328.

## Supplementary Materials

The following supporting information can be downloaded at: https://www.mdpi.com/article/10.3390/life13020347/s1. Figure S1: Representative electron micrograph of serum EVs; Figure S2: Regression lines and correlation coefficients between number of EVs per/mL at the NTA; Figure S3: Verification of serum EV miRNA expression levels evaluated by RT-qPCR; Table S1: Candidate miRNAs for validation set; Table S2: Primers in the present study involved; Table S3: Sensitivity and Specificity of clinical indicators in discriminating between different groups.

## References

[1] Asrani S.K., Hall L., Reddy V., Ogola G., Izzy M. (2022). Comorbid Chronic Diseases and Survival in Compensated and Decompensated Cirrhosis: A Population-Based Study. Off. J. Am. Coll. Gastroenterol. | ACG.

[2] Hu L., Zhu Y., Zhang J., Chen W., Li Z., Li L., Zhang L., Cao D. (2019). Potential Circulating Biomarkers of Circulating Chemokines CCL5, MIP-1β and HA as for Early Detection of Cirrhosis Related to Chronic HBV (Hepatitis B Virus) Infection. BMC Infect. Dis..

[3] Lampertico P., Agarwal K., Berg T., Buti M., Janssen H.L.A., Papatheodoridis G., Zoulim F., Tacke F. (2017). EASL 2017 Clinical Practice Guidelines on the Management of Hepatitis B Virus Infection. J. Hepatol..

[4] European Association for the Study of the Liver (2012). EASL Clinical Practice Guidelines: Management of Chronic Hepatitis B Virus Infection. J. Hepatol..

[5] Tsochatzis E.A., Bosch J., Burroughs A.K. (2014). Liver Cirrhosis. Lancet.

[6] Marcellin P., Kutala B.K. (2018). Liver Diseases: A Major, Neglected Global Public Health Problem Requiring Urgent Actions and Large-Scale Screening. Liver Int..

[7] (2015). World Health Organisation Guideline for the Prevention, Care and Treatment of Persons with Chronic Hepatitis B Infection.

[8] Sonneveld M.J., Brouwer W.P., Chan H.L.-Y., Piratvisuth T., Jia J.-D., Zeuzem S., Liaw Y.-F., Hansen B.E., Choi H., Wat C. (2019). Optimisation of the Use of APRI and FIB-4 to Rule out Cirrhosis in Patients with Chronic Hepatitis B: Results from the SONIC-B Study. Lancet Gastroenterol. Hepatol..

[9] Calvopina D.A., Coleman M.A., Lewindon P.J., Ramm G.A. (2016). Function and Regulation of MicroRNAs and Their Potential as Biomarkers in Paediatric Liver Disease. Int. J. Mol. Sci..

[10] Bedossa P., Dargère D., Paradis V. (2003). Sampling Variability of Liver Fibrosis in Chronic Hepatitis C. Hepatology.

[11] Yang G., Zhuang L., Sun T., Yeo Y.H., Tao L., Zhang W., Ma W., Wu L., Yang Z., Yang Y. (2021). Serum Glial Cell Line-Derived Neurotrophic Factor (SGDNF) Is a Novel Biomarker in Predicting Cirrhosis in Patients with Chronic Hepatitis B. 2021, 2022, 1048104. Can. J. Gastroenterol. Hepatol..

[12] Poynard T., de Ledinghen V., Zarski J.P., Stanciu C., Munteanu M., Vergniol J., France J., Trifan A., Le Naour G., Vaillant J.C. (2012). Relative Performances of FibroTest, Fibroscan, and Biopsy for the Assessment of the Stage of Liver Fibrosis in Patients with Chronic Hepatitis C: A Step toward the Truth in the Absence of a Gold Standard. J. Hepatol..

[13] Imajo K., Kessoku T., Honda Y., Tomeno W., Ogawa Y., Mawatari H., Fujita K., Yoneda M., Taguri M., Hyogo H. (2016). Magnetic Resonance Imaging More Accurately Classifies Steatosis and Fibrosis in Patients with Nonalcoholic Fatty Liver Disease than Transient Elastography. Gastroenterology.

[14] Chen T., Wang C., Yu H., Ding M., Zhang C., Lu X., Zhang C.-Y., Zhang C. (2019). Increased Urinary Exosomal MicroRNAs in Children with Idiopathic Nephrotic Syndrome. EBioMedicine.

[15] Ratajczak J., Miekus K., Kucia M., Zhang J., Reca R., Dvorak P., Ratajczak M.Z. (2006). Embryonic Stem Cell-Derived Microvesicles Reprogram Hematopoietic Progenitors: Evidence for Horizontal Transfer of MRNA and Protein Delivery. Leukemia.

[16] Théry C., Witwer K.W., Aikawa E., Alcaraz M.J., Anderson J.D., Andriantsitohaina R., Antoniou A., Arab T., Archer F., Atkin-Smith G.K. (2018). Minimal Information for Studies of Extracellular Vesicles 2018 (MISEV2018): A Position Statement of the International Society for Extracellular Vesicles and Update of the MISEV2014 Guidelines. J. Extracell. Vesicles.

[17] Quesenberry P.J., Aliotta J.M., Deregibus M.C., Camussi G. (2015). Role of Extracellular RNA-Carrying Vesicles in Cell Differentiation and Reprogramming. Stem Cell Res. Ther..

[18] de Paredes A.G.G., Manicardi N., Tellez L., Ibañez L., Royo F., Bermejo J., Blanco C., Fondevila C., Lanza V.F., Garcia-Bermejo L. (2021). Molecular Profiling of Decompensated Cirrhosis by a Novel MicroRNA Signature. Hepatol. Commun..

[19] Lemoinne S., Thabut D., Housset C., Moreau R., Valla D., Boulanger C.M., Rautou P.-E. (2014). The Emerging Roles of Microvesicles in Liver Diseases. Nat. Rev. Gastroenterol. Hepatol..

[20] Thietart S., Rautou P.-E. (2020). Extracellular Vesicles as Biomarkers in Liver Diseases: A Clinician’s Point of View. J. Hepatol..

[21] Kornek M., Lynch M., Mehta S.H., Lai M., Exley M., Afdhal N.H., Schuppan D. (2012). Circulating Microparticles as Disease-Specific Biomarkers of Severity of Inflammation in Patients with Hepatitis C or Nonalcoholic Steatohepatitis. Gastroenterology.

[22] Rautou P.-E., Bresson J., Sainte-Marie Y., Vion A.-C., Paradis V., Renard J., Devue C., Heymes C., Letteron P., Elkrief L. (2012). Abnormal Plasma Microparticles Impair Vasoconstrictor Responses in Patients With Cirrhosis. Gastroenterology.

[23] Payancé A., Silva-Junior G., Bissonnette J., Tanguy M., Pasquet B., Levi C., Roux O., Nekachtali O., Baiges A., Hernández-Gea V. (2018). Hepatocyte Microvesicle Levels Improve Prediction of Mortality in Patients with Cirrhosis. Hepatology.

[24] Campello E., Zanetto A., Spiezia L., Radu C.M., Gavasso S., Ferrarese A., Farinati F., Senzolo M., Simioni P. (2016). Hypercoagulability Detected by Circulating Microparticles in Patients with Hepatocellular Carcinoma and Cirrhosis. Thromb. Res..

[25] Friedman R.C., Farh K.K.-H., Burge C.B., Bartel D.P. (2009). Most Mammalian MRNAs Are Conserved Targets of MicroRNAs. Genome Res..

[26] Diehl P., Fricke A., Sander L., Stamm J., Bassler N., Htun N., Ziemann M., Helbing T., El-Osta A., Jowett J.B.M. (2012). Microparticles: Major Transport Vehicles for Distinct MicroRNAs in Circulation. Cardiovasc. Res..

[27] Vallabhajosyula P., Korutla L., Habertheuer A., Yu M., Rostami S., Yuan C.X., Reddy S., Liu C., Korutla V., Koeberlein B. (2017). Tissue-Specific Exosome Biomarkers for Noninvasively Monitoring Immunologic Rejection of Transplanted Tissue. J. Clin. Investig..

[28] Habertheuer A., Korutla L., Rostami S., Reddy S., Lal P., Naji A., Vallabhajosyula P. (2018). Donor Tissue-Specific Exosome Profiling Enables Noninvasive Monitoring of Acute Rejection in Mouse Allogeneic Heart Transplantation. J. Thorac. Cardiovasc. Surg..

[29] Szabo G., Bala S. (2013). MicroRNAs in Liver Disease. Nat. Rev. Gastroenterol. Hepatol..

[30] Farid W.R.R., Pan Q., van der Meer A.J.P., de Ruiter P.E., Ramakrishnaiah V., de Jonge J., Kwekkeboom J., Janssen H.L.A., Metselaar H.J., Tilanus H.W. (2012). Hepatocyte-Derived MicroRNAs as Serum Biomarkers of Hepatic Injury and Rejection after Liver Transplantation. Liver Transplant..

[31] Ohno S., Ishikawa A., Kuroda M. (2013). Roles of Exosomes and Microvesicles in Disease Pathogenesis. Adv. Drug Deliv. Rev..

[32] Lee Y.-S., Kim S.Y., Ko E., Lee J.-H., Yi H.-S., Yoo Y.J., Je J., Suh S.J., Jung Y.K., Kim J.H. (2017). Exosomes Derived from Palmitic Acid-Treated Hepatocytes Induce Fibrotic Activation of Hepatic Stellate Cells. Sci. Rep..

[33] Pirola C.J., Gianotti T.F., Castaño G.O., Mallardi P., San Martino J., Ledesma M.M.G.L., Flichman D., Mirshahi F., Sanyal A.J., Sookoian S. (2015). Circulating MicroRNA Signature in Non-Alcoholic Fatty Liver Disease: From Serum Non-Coding RNAs to Liver Histology and Disease Pathogenesis. Gut.

[34] Akuta N., Kawamura Y., Watanabe C., Nishimura A., Okubo M., Mori Y., Fujiyama S., Sezaki H., Hosaka T., Kobayashi M. (2019). Impact of Sodium Glucose Cotransporter 2 Inhibitor on Histological Features and Glucose Metabolism of Non-alcoholic Fatty Liver Disease Complicated by Diabetes Mellitus. Hepatol. Res..

[35] Wang H., Hou L., Li A., Duan Y., Gao H., Song X. (2014). Expression of Serum Exosomal MicroRNA-21 in Human Hepatocellular Carcinoma. Biomed Res. Int..

[36] Xue X., Zhao Y., Wang X., Qin L., Hu R. (2019). Development and Validation of Serum Exosomal MicroRNAs as Diagnostic and Prognostic Biomarkers for Hepatocellular Carcinoma. J. Cell. Biochem..

[37] Chinese Society of Infectious Diseases, Chinese Society of Hepatology (2019). The Guidelines of Prevention and Treatment for Chronic Hepatitis B (2019 Version). Zhonghua Gan Zang Bing Za Zhi = Zhonghua Ganzangbing Zazhi = Chin. J. Hepatol..

[38] Langmead B., Trapnell C., Pop M., Salzberg S.L. (2009). Ultrafast and Memory-Efficient Alignment of Short DNA Sequences to the Human Genome. Genome Biol..

[39] Friedländer M.R., Chen W., Adamidi C., Maaskola J., Einspanier R., Knespel S., Rajewsky N. (2008). Discovering MicroRNAs from Deep Sequencing Data Using MiRDeep. Nat. Biotechnol..

[40] Nawrocki E.P., Eddy S.R. (2013). Infernal 1.1: 100-Fold Faster RNA Homology Searches. Bioinformatics.

[41] Callewaert N., Van Vlierberghe H., Van Hecke A., Laroy W., Delanghe J., Contreras R. (2004). Noninvasive Diagnosis of Liver Cirrhosis Using DNA Sequencer–Based Total Serum Protein Glycomics. Nat. Med..

[42] Chen Y.-J., Zhu J.-M., Wu H., Fan J., Zhou J., Hu J., Yu Q., Liu T.-T., Yang L., Wu C.-L. (2013). Circulating MicroRNAs as a Fingerprint for Liver Cirrhosis. PLoS ONE.

[43] Xie Y., Yao Q., Butt A.M., Guo J., Tian Z., Bao X., Li H., Meng Q., Lu J. (2014). Expression Profiling of Serum MicroRNA-101 in HBV-Associated Chronic Hepatitis, Liver Cirrhosis, and Hepatocellular Carcinoma. Cancer Biol. Ther..

[44] Mölleken C., Sitek B., Henkel C., Poschmann G., Sipos B., Wiese S., Warscheid B., Broelsch C., Reiser M., Friedman S.L. (2009). Detection of Novel Biomarkers of Liver Cirrhosis by Proteomic Analysis. Hepatology.

[45] Guo H., Ingolia N.T., Weissman J.S., Bartel D.P. (2010). Mammalian MicroRNAs Predominantly Act to Decrease Target MRNA Levels. Nature.

[46] Sohn W., Kim J., Kang S.H., Yang S.R., Cho J.-Y., Cho H.C., Shim S.G., Paik Y.-H. (2015). Serum Exosomal MicroRNAs as Novel Biomarkers for Hepatocellular Carcinoma. Exp. Mol. Med..

[47] Csak T., Bala S., Lippai D., Satishchandran A., Catalano D., Kodys K., Szabo G. (2015). MicroRNA-122 Regulates Hypoxia-Inducible Factor-1 and Vimentin in Hepatocytes and Correlates with Fibrosis in Diet-Induced Steatohepatitis. Liver Int..

[48] Cho H.J., Eun J.W., Baek G.O., Seo C.W., Ahn H.R., Kim S.S., Cho S.W., Cheong J.Y. (2020). Serum Exosomal MicroRNA, MiR-10b-5p, as a Potential Diagnostic Biomarker for Early-Stage Hepatocellular Carcinoma. J. Clin. Med..

[49] Saviano A., Henderson N.C., Baumert T.F. (2020). Single-Cell Genomics and Spatial Transcriptomics: Discovery of Novel Cell States and Cellular Interactions in Liver Physiology and Disease Biology. J. Hepatol..

[50] Daemen S., Gainullina A., Kalugotla G., He L., Chan M.M., Beals J.W., Liss K.H., Klein S., Feldstein A.E., Finck B.N. (2021). Dynamic Shifts in the Composition of Resident and Recruited Macrophages Influence Tissue Remodeling in NASH. Cell Rep..

[51] Colombo M., Moita C., van Niel G., Kowal J., Vigneron J., Benaroch P., Manel N., Moita L.F., Théry C., Raposo G. (2013). Analysis of ESCRT functions in exosome biogenesis, composition and secretion highlights the heterogeneity of extracellular vesicles. J. Cell Sci..

[52] Logozzi M., Mizzoni D., Di Raimo R., Giuliani A., Maggi M., Sciarra A., Fais S. (2021). Plasmatic Exosome Number and Size Distinguish Prostate Cancer Patients From Healthy Individuals: A Prospective Clinical Study. Front. Oncol..

[53] Burrello J., Gai C., Tetti M., Lopatina T., Deregibus M.C., Veglio F., Mulatero P., Camussi G., Monticone S. (2019). Characterization and Gene Expression Analysis of Serum-Derived Extracellular Vesicles in Primary Aldosteronism. Hypertension.

[54] Bernal-Mizrachi L., Jy W., Jimenez J.J., Pastor J., Mauro L.M., Horstman L.L., De Marchena E., Ahn Y.S. (2003). High Levels of Circulating Endothelial Microparticles in Patients with Acute Coronary Syndromes. Am. Heart J..

[55] Amabile N., Guérin A.P., Leroyer A., Mallat Z., Nguyen C., Boddaert J., London G.M., Tedgui A., Boulanger C.M. (2005). Circulating Endothelial Microparticles Are Associated with Vascular Dysfunction in Patients with End-Stage Renal Failure. J. Am. Soc. Nephrol..

[56] Sabatier F., Darmon P., Hugel B., Combes V., Sanmarco M., Velut J.-G., Arnoux D., Charpiot P., Freyssinet J.-M., Oliver C. (2002). Type 1 And Type 2 Diabetic Patients Display Different Patterns of Cellular Microparticles. Diabetes.

[57] González-Quintero V.H., Jiménez J.J., Jy W., Mauro L.M., Hortman L., O’Sullivan M.J., Ahn Y. (2003). Elevated Plasma Endothelial Microparticles in Preeclampsia. Am. J. Obstet. Gynecol..

[58] Wang L., Wang J., Wang Z., Zhou J., Zhang Y. (2021). Higher Urine Exosomal MiR-193a Is Associated with a Higher Probability of Primary Focal Segmental Glomerulosclerosis and an Increased Risk of Poor Prognosis among Children with Nephrotic Syndrome. Front. Cell Dev. Biol..

[59] Savina A., Furlán M., Vidal M., Colombo M.I. (2003). Exosome Release Is Regulated by a Calcium-Dependent Mechanism in K562 Cells. J. Biol. Chem..

[60] Prudent M., Crettaz D., Delobel J., Seghatchian J., Tissot J.D., Lion N. (2015). Differences between Calcium-Stimulated and Storage-Induced Erythrocyte-Derived Microvesicles. Transfus. Apher. Sci..

[61] Lange S., Gallagher M., Kholia S., Kosgodage U.S., Hristova M., Hardy J., Inal J.M. (2017). Peptidylarginine DeiminasesRoles in Cancer and Neurodegeneration and Possible Avenues for Therapeutic Intervention via Modulation of Exosome and Microvesicle (EMV) Release?. Int. J. Mol. Sci..

[62] Choi D.-Y., Park J.-N., Paek S.-H., Choi S.-C., Paek S.-H. (2021). Detecting Early-Stage Malignant Melanoma Using a Calcium Switch-Enriched Exosome Subpopulation Containing Tumor Markers as a Sample. Biosens. Bioelectron..

[63] Bucki R., Bachelot-Loza C., Zachowski A., Giraud F., Sulpice J.C. (1998). Calcium Induces Phospholipid Redistribution and Microvesicle Release in Human Erythrocyte Membranes by Independent Pathways. Biochemistry.

[64] Draeger A., Schoenauer R., Atanassoff A.P., Wolfmeier H., Babiychuk E.B. (2014). Dealing with Damage: Plasma Membrane Repair Mechanisms. Biochimie.

[65] Luo W.-J., Cheng T.-Y., Wong K.-I., Fang W., Liao K.-M., Hsieh Y.-T., Su K.-Y. (2018). Novel Therapeutic Drug Identification and Gene Correlation for Fatty Liver Disease Using High-Content Screening: Proof of Concept. Eur. J. Pharm. Sci..

[66] Zhao X., Fan H., Chen X., Zhao X., Wang X., Feng Y., Liu M., Li S., Tang H. (2021). Hepatitis B Virus DNA Polymerase Restrains Viral Replication Through the CREB1/HOXA Distal Transcript Antisense RNA Homeobox A13 Axis. Hepatology.

[67] Allan G.J., Beattie J., Flint D.J. (2008). Epithelial Injury Induces an Innate Repair Mechanism Linked to Cellular Senescence and Fibrosis Involving IGF-Binding Protein-5. J. Endocrinol..

[68] Lecca M.R., Maag C., Berger E.G., Hennet T. (2011). Fibrotic Response in Fibroblasts from Congenital Disorders of Glycosylation. J. Cell. Mol. Med..

[69] Hironaka-Mitsuhashi A., Otsuka K., Gailhouste L., Calle A.S., Kumazaki M., Yamamoto Y., Fujiwara Y., Ochiya T. (2020). MiR-1285-5p/TMEM194A Axis Affects Cell Proliferation in Breast Cancer. Cancer Sci..

[70] Wang X., Yan M., Zhao L., Wu Q., Wu C., Chang X., Zhou Z. (2016). Low-Dose Methylmercury-Induced Genes Regulate Mitochondrial Biogenesis via MiR-25 in Immortalized Human Embryonic Neural Progenitor Cells. Int. J. Mol. Sci..

[71] Villanova L., Barbini C., Piccolo C., Boe A., De Maria R., Fiori M.E. (2020). MiR-1285-3p Controls Colorectal Cancer Proliferation and Escape from Apoptosis through DAPK2. Int. J. Mol. Sci..

[72] Pao S.I., Lin L.T., Chen Y.H., Chen C.L., Chen J.T. (2021). Repression of Smad4 by MicroRNA-1285 Moderates TGF-β-Induced Epithelial–Mesenchymal Transition in Proliferative Vitreoretinopathy. PLoS ONE.

[73] Sun X., Lin F., Sun W., Zhu W., Fang D., Luo L., Li S., Zhang W., Jiang L. (2021). Exosome-Transmitted MiRNA-335-5p Promotes Colorectal Cancer Invasion and Metastasis by Facilitating EMT via Targeting RASA1. Mol. Ther. Nucleic Acids.

[74] Sun J., Lu H., Liang W., Zhao G., Ren L., Hu D., Chang Z., Liu Y., Garcia-Barrio M.T., Zhang J. (2021). Endothelial TFEB (Transcription Factor EB) Improves Glucose Tolerance via Upregulation of IRS (Insulin Receptor Substrate) 1 and IRS2. Arterioscler. Thromb. Vasc. Biol..

