# Supplementary material for: Characterization and microRNA Expression Analysis of Serum-Derived Extracellular Vesicles in Severe Liver Injury from Chronic HBV Infection
Source: Life (Basel). 2023 Jan 28;13(2):347. doi: 10.3390/life13020347 (PMC9967308; doi:10.3390/life13020347)

SUPPLEMENTAL MATERIAL

SUPPLEMENTAL TABLE S1. Candidate miRNAs for validation set.

Gene ID	log2 (FoldChange)	Qvalue	Regulation
hsa-miR-122-3p	4.83	4.93E-210	Up
hsa-miR-4433a-3p	3.62	9.90E-60	Up
hsa-miR-877-5p	2.95	1.10E-06	Up
hsa-miR-1468-5p	2.6	4.17E-74	Up
hsa-miR-29a-3p	-3.13	1.95E-168	Down
hsa-miR-29c-3p	-3.62	1.93E-105	Down
novel-hsa-miR172-5p	-4.14	1.49E-68	Down
hsa-miR-411-5p	-4.18	1.10E-121	Down
hsa-miR-379-5p	-4.87	5.62E-151	Down
hsa-miR-1-3p	-6.62	0.00E+00	Down
hsa-miR-133a-3p	-7.4	0.00E+00	Down
hsa-miR-335-5p	-8.14	3.09E-08	Down
hsa-miR-204-3p	-8.64	3.43E-11	Down
hsa-miR-1285-5p	-8.75	7.70E-13	Down
hsa-miR-206	-10.01	0.00E+00	Down

SUPPLEMENTAL TABLE S2. Primers in the present study involved.

miRNAs	RT Primer(5'-3')	Forward Primer(5'-3')
cel-miR-39-3p	GTCGTATCCAGTGCAGGGTCCGAGGTATTCGCACTGGATACGACCAAGCT	GCGTCACCGGGTGTAAATC
novel-hsa-miR-172-5p	GTCGTATCCAGTGCAGGGTCCGAGGTATTCGCACTGGATACGACCGCCAC	CGCGAGGCTGGAGTGCA
hsa-miR-122-3p	GTCGTATCCAGTGCAGGGTCCGAGGTATTCGCACTGGATACGACTATTTA	CGCGAACGCCATTATCACAC
hsa-miR-1468-5p	GTCGTATCCAGTGCAGGGTCCGAGGTATTCGCACTGGATACGACCAGCGA	GCGCTCCGTTTGCCTGTT
hsa-miR-1-3p	GTCGTATCCAGTGCAGGGTCCGAGGTATTCGCACTGGATACGACATACAT	GCGCGTGGAATGTAAAGAAGT
hsa-miR-411-5p	GTCGTATCCAGTGCAGGGTCCGAGGTATTCGCACTGGATACGACCGTACG	CGCGCGTAGTAGACCGTATAG
hsa-miR-29c-3p	GTCGTATCCAGTGCAGGGTCCGAGGTATTCGCACTGGATACGACTAACCG	CGCGTAGCACCATTTGAAAT
hsa-miR-133a-3p	GTCGTATCCAGTGCAGGGTCCGAGGTATTCGCACTGGATACGACCAGCTG	GCGTTTGGTCCCCTTCAAC
hsa-miR-29a-3p	GTCGTATCCAGTGCAGGGTCCGAGGTATTCGCACTGGATACGACTAACCG	CGCGTAGCACCATCTGAAAT
hsa-miR-204-3p	GTCGTATCCAGTGCAGGGTCCGAGGTATTCGCACTGGATACGACCAGCTG	GCGTTTGGTCCCCTTCAAC
hsa-miR-206	GTCGTATCCAGTGCAGGGTCCGAGGTATTCGCACTGGATACGACCCACAC	GCGCGTGGAATGTAAGGAAGT
hsa-miR-1285-5p	GTCGTATCCAGTGCAGGGTCCGAGGTATTCGCACTGGATACGACCCTGGG	GCGCGGATCTCACTTTGTTG
hsa-miR-335-5p	GTCGTATCCAGTGCAGGGTCCGAGGTATTCGCACTGGATACGACACATTT	CGCGTCAAGAGCAATAACGAA
hsa-miR-877-5p	GTCGTATCCAGTGCAGGGTCCGAGGTATTCGCACTGGATACGACCCCTGC	CGCGGTAGAGGAGATGGC
hsa-miR-379-5p	GTCGTATCCAGTGCAGGGTCCGAGGTATTCGCACTGGATACGACCCTACG	GCGCGTGGTAGACTATGGAA
hsa-miR-4433a-3p	GTCGTATCCAGTGCAGGGTCCGAGGTATTCGCACTGGATACGACATGTCC	GCGACAGGAGTGGGGGTG

SUPPLEMENTAL TABLE S3. Sensitivity and Specificity of clinical indicators in discriminating between different groups.

Group	Target	Sensitivity%	Specificity%
NC vs. CHB	novel-has-miR-172-5p	84.85	72.73
	has-miR-1285-5p	79.17	71.43
	miRNA panel[1]	83.33	76.19
NC vs. DeCi	novel-has-miR-172-5p	69.70	75.76
	has-miR-1285-5p	58.33	95.24
	has-miR-335-5p	85.29	58.06
	miRNA panel[2]	83.33	80.95
CHB vs. DeCi	has-miR-335-5p	88.24	41.18
	CHE	86.49	86.84
	AST/ALT	82.69	87.72
	Ca^{2+}	89.83	77.36
	panel[3]	-	-
	panel[4]	91.67	91.30

miRNA panel[1] is the combination of miR-172-5p and miR-1285-5p;

miRNA panel[2] is the combination of miR-172-5p, miR-1285-5p, and miR-335-5p;

panel[3] is the combination of AST/ALT, Ca^{2+}, and CHE;

panel[4] is the combination of AST/ALT, Ca^{2+}, CHE, and miR-335-5p.

SUPPLEMENTAL FIGURE S1. Representative electron micrograph of serum EVs. Scale bar=100 nm.

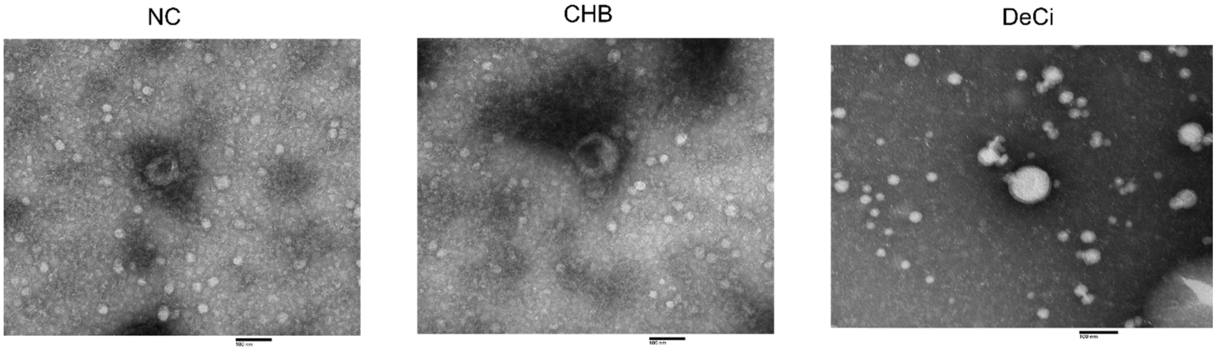

SUPPLEMENTAL FIGURE S2. Regression lines and correlation coefficients between number of EVs per/mL at the NTA. (**A**) Quantification of nanoparticles using NTA. (**B**) Diameter of EVs in nm using NTA. In **A** and **B**, the horizontal line indicates the median and box, and bars represent the 25–75th and the 5–95th percentiles, respectively. ns, not significant.

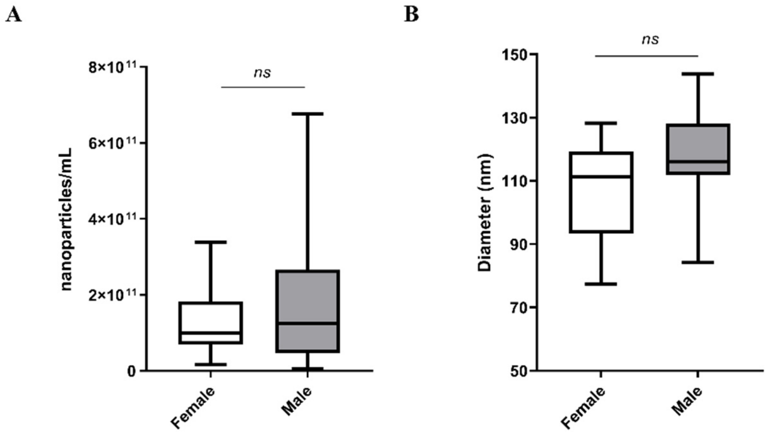

SUPPLEMENTAL FIGURE S3. Verification of serum EV miRNA expression levels evaluated by qRT-PCR. (A–C) The expression profile of serum EV hsa-miR-411-5p (A), hsa-miR-877-5p (B), and hsa-miR-4433-5p (C) in NC, CHB, and DeCi patients. Each P value was calculated with a nonparametric Mann–Whitney test: ns, not significant.

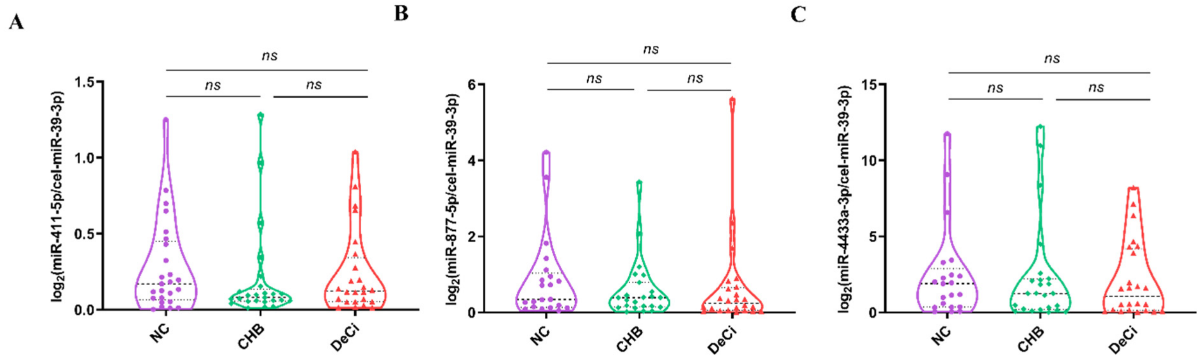

Supplement: Supplementary file 1 [file life-13-00347-s001.zip › supplemantary_Characterization_and_microRNA.pdf]
