# Supplementary figures and images for: Characterization and microRNA Expression Analysis of Serum-Derived Extracellular Vesicles in Severe Liver Injury from Chronic HBV Infection
Source: Life (Basel). 2023 Jan 28;13(2):347. doi: 10.3390/life13020347 (PMC9967308; doi:10.3390/life13020347)

NC

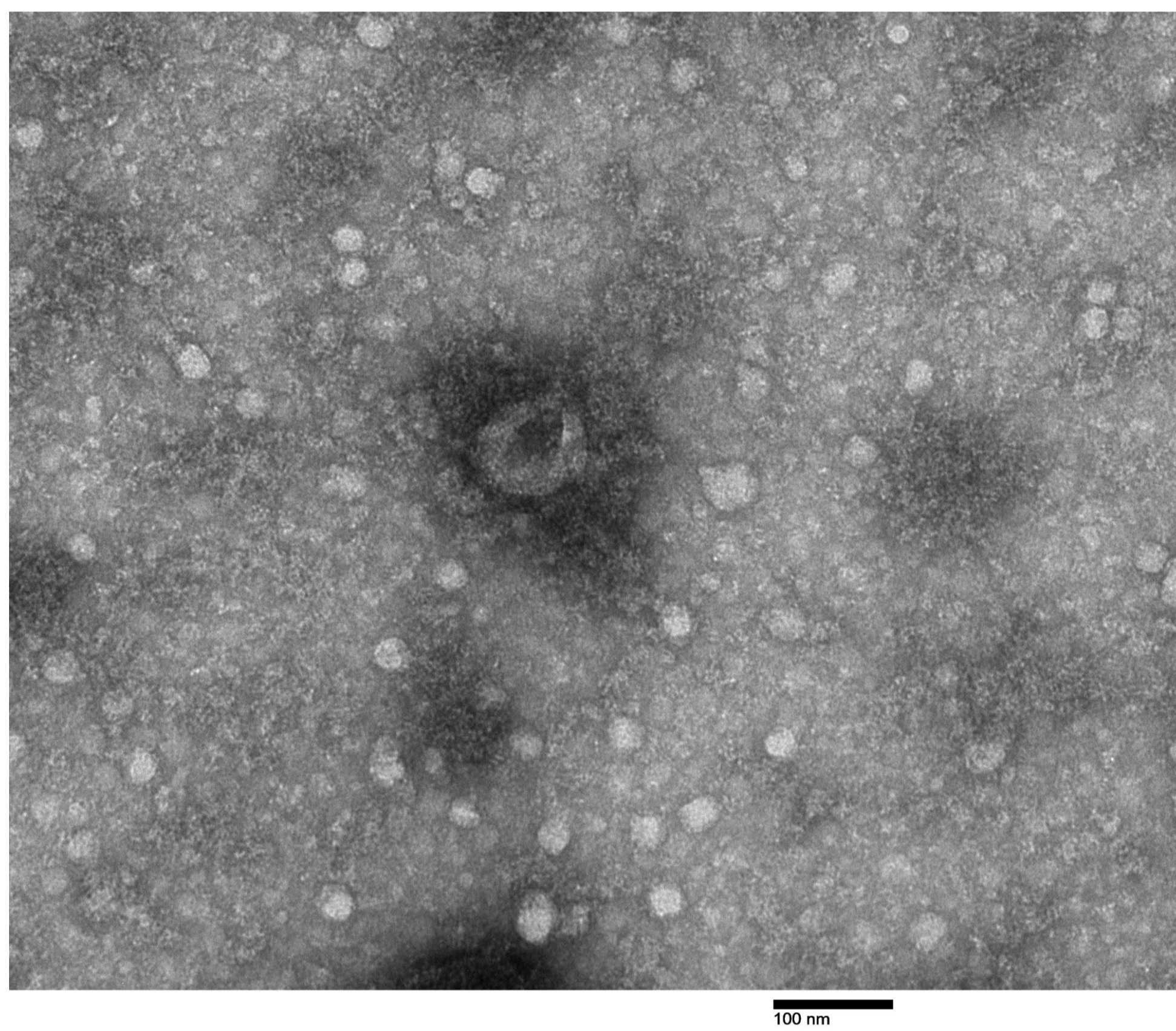

CHB

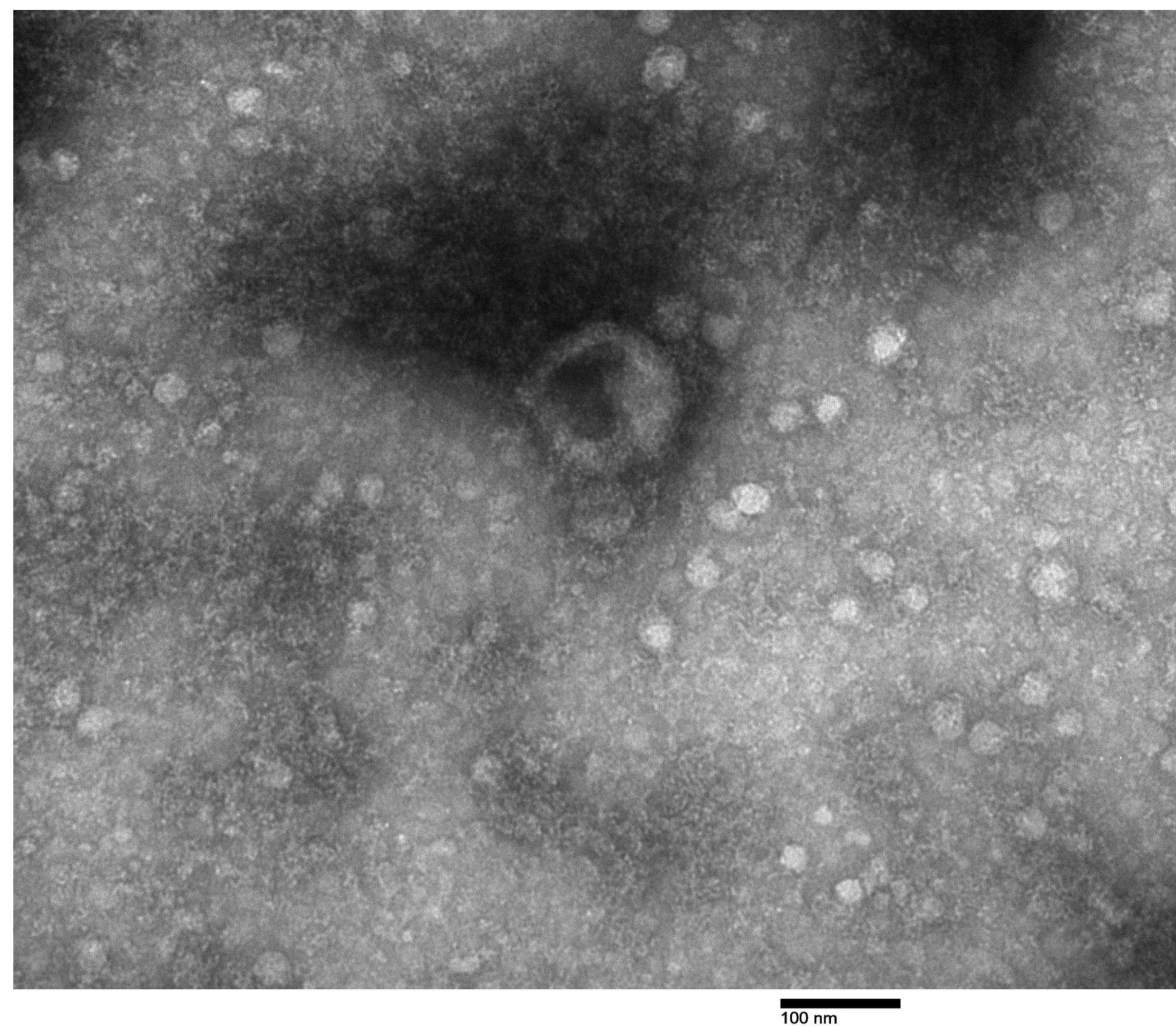

DeCi

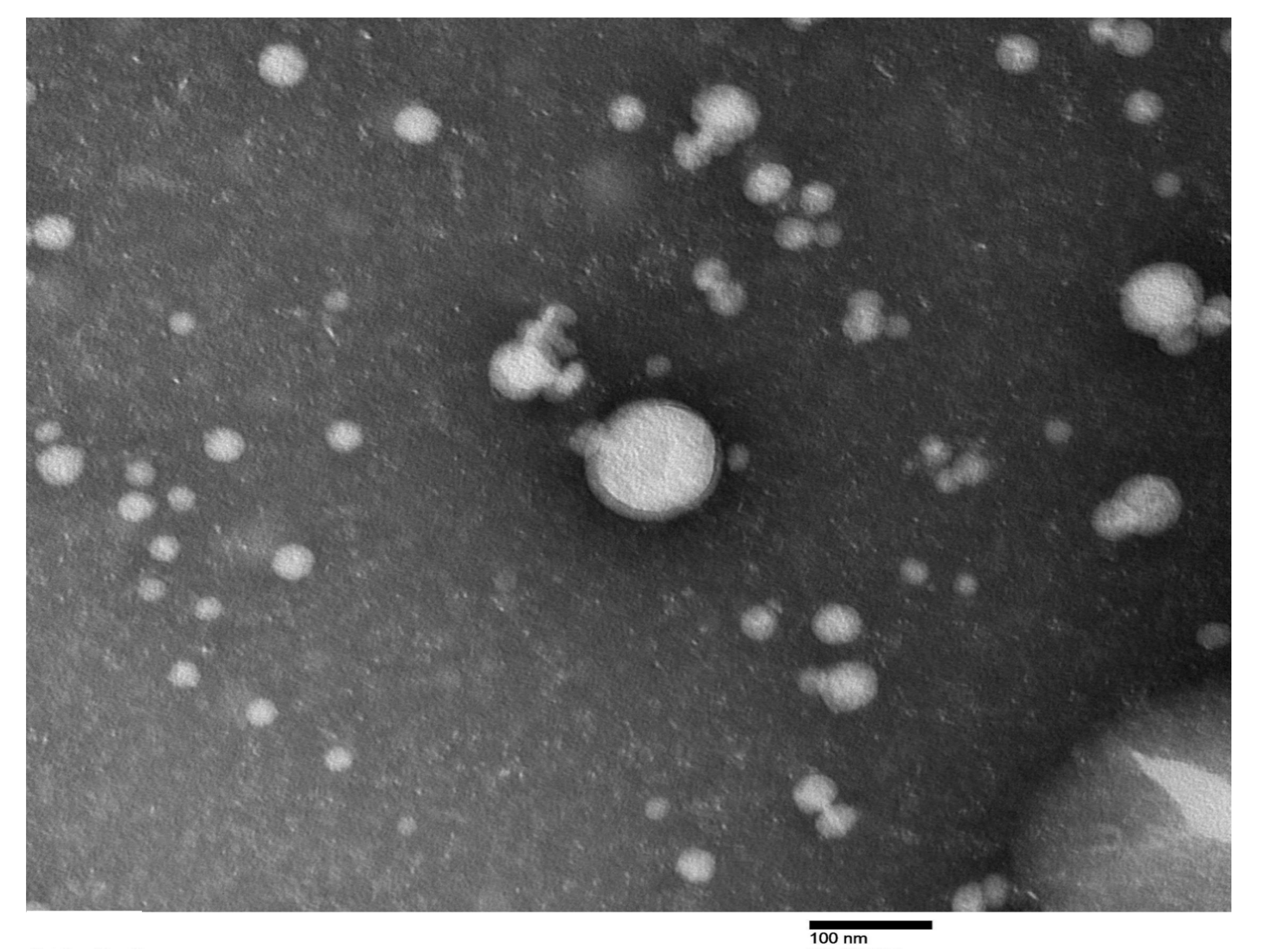

Supplement: Supplementary file 1 [file life-13-00347-s001.zip › figure.S1_R.pdf]

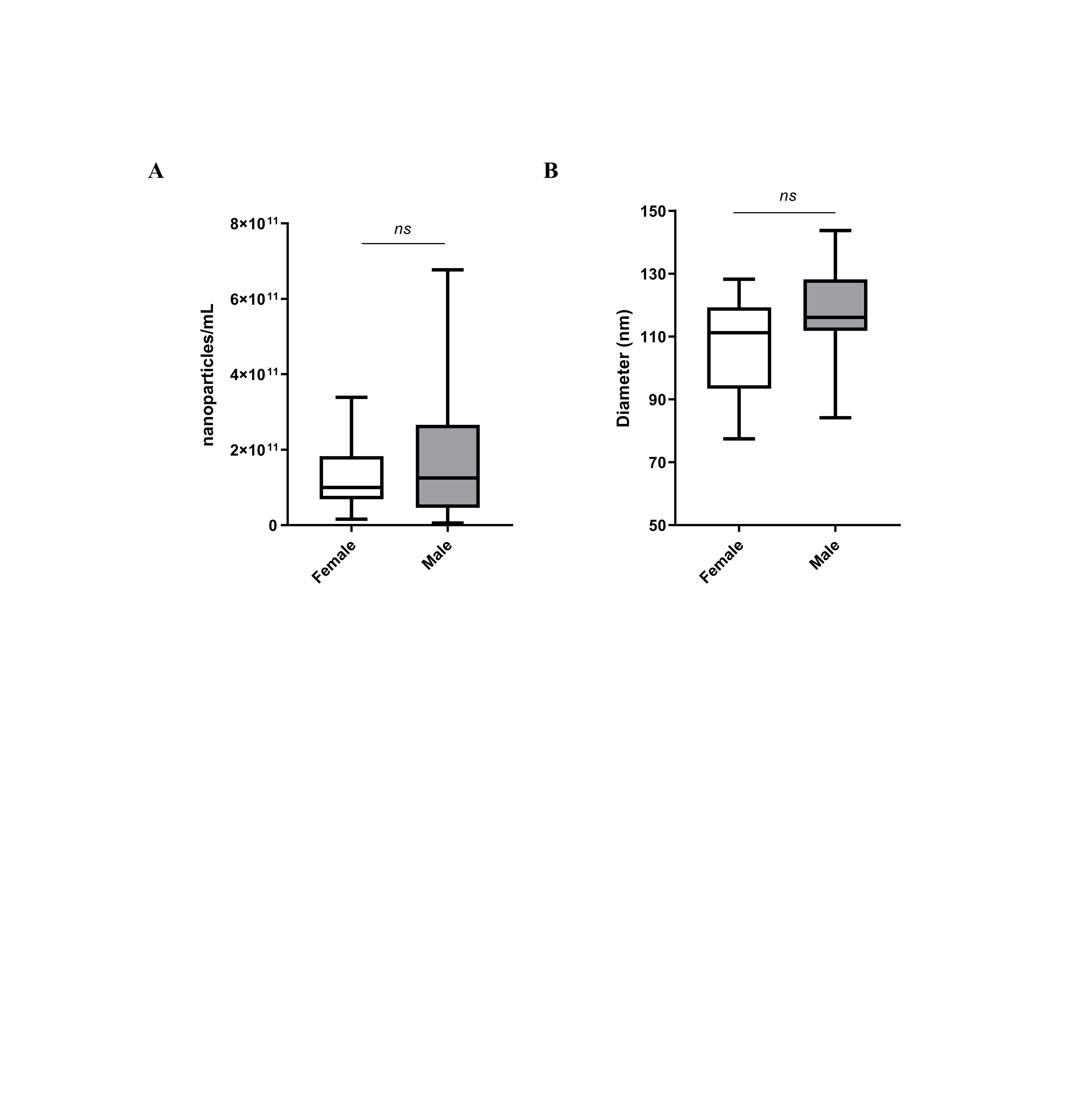

Supplement: Supplementary file 1 [file life-13-00347-s001.zip › figure.S2.tif]

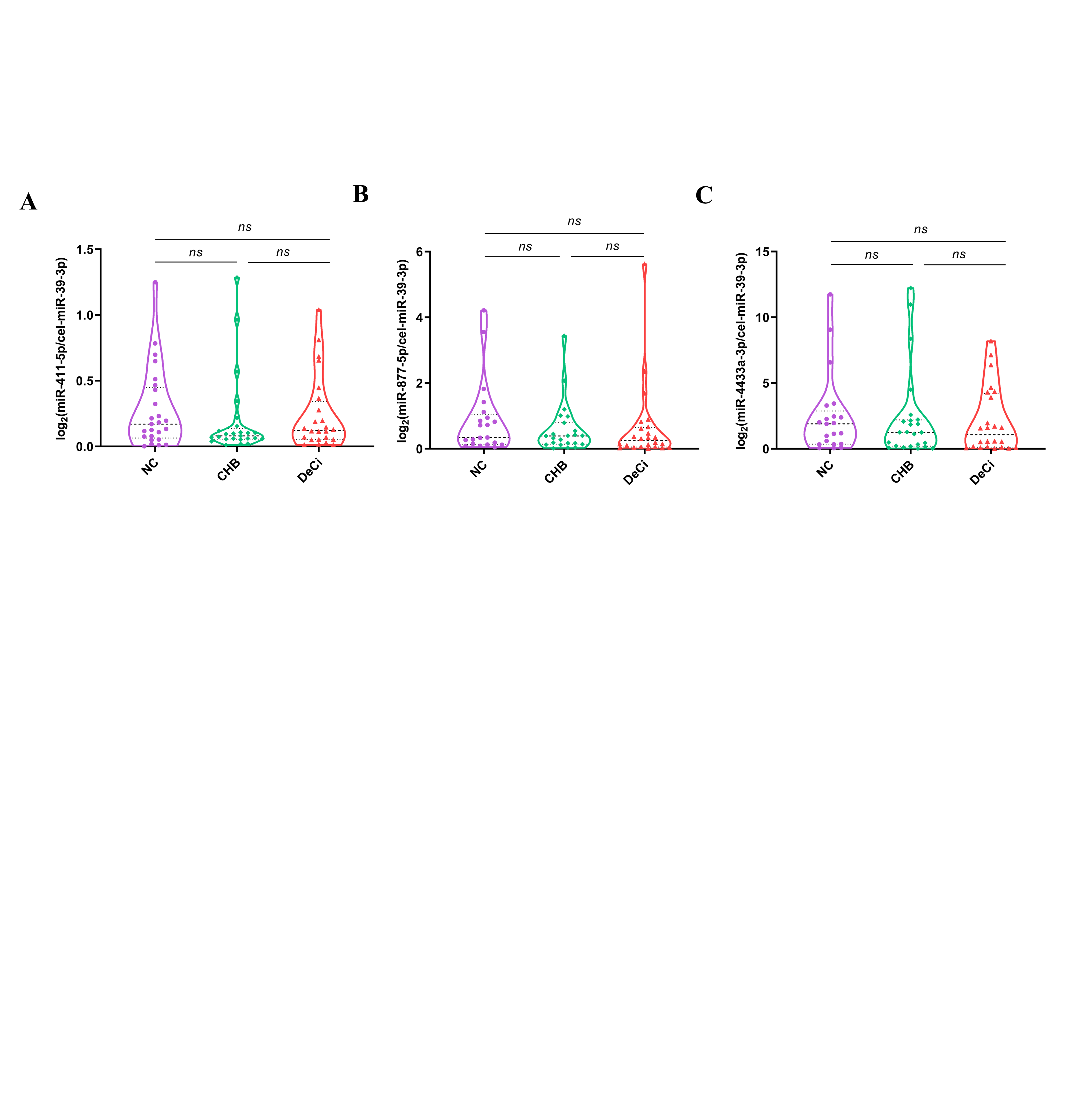

Supplement: Supplementary file 1 [file life-13-00347-s001.zip › figure.S3.tif]
